# Do antidepressants increase the risk of mania and bipolar disorder in people with depression? A retrospective electronic case register cohort study

**DOI:** 10.1136/bmjopen-2015-008341

**Published:** 2015-12-01

**Authors:** Rashmi Patel, Peter Reiss, Hitesh Shetty, Matthew Broadbent, Robert Stewart, Philip McGuire, Matthew Taylor

**Affiliations:** 1 Department of Psychosis Studies, King's College London, Institute of Psychiatry, Psychology & Neuroscience, London, UK; 2 Biomedical Research Centre Nucleus, South London and Maudsley NHS Foundation Trust, London, UK; 3 Department of Psychological Medicine, King's College London, Institute of Psychiatry, Psychology & Neuroscience, London, UK

**Keywords:** antidepressant induced mania, manic switch, CRIS, electronic health records, SSRI, venlafaxine

## Abstract

**Objectives:**

To investigate the association between antidepressant therapy and the later onset of mania/bipolar disorder.

**Design:**

Retrospective cohort study using an anonymised electronic health record case register.

**Setting:**

South London and Maudsley National Health Service (NHS) Trust (SLaM), a large provider of inpatient and community mental healthcare in the UK.

**Participants:**

21 012 adults presenting to SLaM between 1 April 2006 and 31 March 2013 with unipolar depression.

**Exposure:**

Prior antidepressant therapy recorded in electronic health records.

**Main outcome measure:**

Time to subsequent diagnosis of mania or bipolar disorder from date of diagnosis of unipolar depression, censored at 31 March 2014.

**Methods:**

Multivariable Cox regression analysis with age and gender as covariates.

**Results:**

The overall incidence rate of mania/bipolar disorder was 10.9 per 1000 person-years. The peak incidence of mania/bipolar disorder incidence was seen in patients aged between 26 and 35 years (12.3 per 1000 person-years). Prior antidepressant treatment was associated with an increased incidence of mania/bipolar disorder ranging from 13.1 to 19.1 per 1000 person-years. Multivariable analysis indicated a significant association with selective serotonin reuptake inhibitors (HR 1.34, 95% CI 1.18 to 1.52) and venlafaxine (1.35, 1.07 to 1.70).

**Conclusions:**

In people with unipolar depression, antidepressant treatment is associated with an increased risk of subsequent mania/bipolar disorder. These findings highlight the importance of considering risk factors for mania when treating people with depression.

**Strengths and limitations of this study**

- The findings were drawn from a large population (over 21 000 adults) using data from electronic health records which are representative of everyday clinical practice. The results are therefore generally applicable.
- Because it was an observational study, it is not possible to infer a causal link between antidepressant treatment and an increased incidence of mania or bipolar disorder.
- Although the findings are based on data recorded when patients were receiving secondary mental healthcare, most will have originally been diagnosed with depression and initially treated in primary care. Some of these patients may have developed mania prior to contact with secondary mental healthcare services, resulting in an underestimate of the incidence of mania/bipolar disorder.

## Introduction

The occurrence of mania and hypomania in people receiving antidepressant therapy is an adverse effect of treatment with antidepressant medication.^1^ However, it is unclear whether antidepressants cause acute mania or hypomania in patients with unipolar depression or trigger the expression of an underlying bipolar disorder.^2^ Acute mania has been particularly associated with TCAs and dual-action antidepressants such as venlafaxine.^1^ ^3^

As patients with bipolar disorder typically present during a depressive or mixed affective episode rather than during a hypomanic or manic episode,^4^ and depressive symptoms tend to dominate the course of the illness,^5^ ^6^ a proportion of patients treated for unipolar depression may have an underlying bipolar disorder.^7^ ^8^

Previous findings exploring this area have been derived from data compiled from a number of studies that differ in type, size and design, and often without the explicit goal of identifying the rate of mania or hypomania in patients with unipolar depression.^1^ An investigation focusing on incidence in a ‘real-world’ sample receiving routine care may give a more meaningful estimation of the association of antidepressants with mania or hypomania. In the present study, we examined the electronic health records of a large sample of patients receiving secondary mental healthcare for unipolar depression. We extracted data on prior antidepressant use and subsequent diagnosis of mania or bipolar disorder, then tested the hypothesis that antidepressant exposure was associated with an increase in incidence of subsequent mania or bipolar disorder.

## Methods

### Participants

We included all individuals aged between 16 and 65 receiving mental healthcare from the South London and Maudsley National Health Service (NHS) Foundation Trust (SLaM) between 1 April 2006 and 31 March 2013 with a diagnosis of depression (International Classification of Diseases (ICD)-10 F32/F33), and no prior diagnosis of mania or bipolar disorder (F30/F31). Applying these inclusion criteria yielded a sample of 21 012 patients. The follow-up period was from the date that depression was first diagnosed to 31 March 2014 and comprised 91 110 person-years with a mean follow-up duration of 4.3 years.

### Source of clinical data

Data were obtained for this study from the SLaM Biomedical Research Centre (BRC) Case Register.^9^ SLaM is a large provider of mental healthcare covering a catchment area of around 1.2 million residents in South London. Clinical records in SLaM have been documented in a single electronic health record system (the electronic Patient Journey System—ePJS) since April 2006. Anonymised clinical data from ePJS including structured fields (for demographic, diagnostic and medication data) and pseudonymised unstructured free-text fields from case notes and correspondence have been extracted into the SLaM BRC Case Register.^10^ Clinical information is documented by healthcare professionals during the course of providing mental healthcare to patients and includes history, mental state examination, diagnostic formulation and management plans. The healthcare professionals who document clinical data include psychiatrists, psychologists, nursing staff, care coordinators and allied healthcare professionals. Diagnostic information is generally recorded by a psychiatrist and is based on clinical interview. Data for this study were obtained from these sources of clinical data in the SLaM BRC Case Register using the Clinical Record Interactive Search tool (CRIS). CRIS is a bespoke database search and assembly tool which has supported a range of studies using this data set.^11–16^ CRIS obtains data from the SLaM BRC Case Register from structured electronic health record fields and also using natural language processing (NLP) from unstructured free-text documentation. In order to maximise ascertainment of diagnosis and prior antidepressant treatment in this study, data on these variables were obtained from both structured fields and unstructured free-text clinical entries (using NLP).^17^

### Ascertainment of prior antidepressant therapy

Prior antidepressant therapy was defined as documentation of antidepressant treatment prior to the date of diagnosis of depression. This definition was chosen on the basis of a previous study which indicated that in the SLaM BRC Case Register, the documentation of treatment for a mental health disorder generally occurs prior to the documentation of a formal diagnosis.^18^ For the purposes of this study, antidepressants were defined as any licensed antidepressant medication listed in section 4.3 of the British National Formulary (BNF)^19^ in the following groups: Monoamine-oxidase inhibitors; mirtazapine; selective serotonin reuptake inhibitors (SSRI); TCAs; trazodone; venlafaxine; duloxetine; other antidepressants (agomelatine and reboxetine). These groups were chosen based on the UK National Institute for Health and Care Excellence (NICE) guideline (CG90) for treatment and management of depression in adults.^20^ Each of these groups was analysed as a binary variable defined as treatment with any drug within each group.

### Clinical outcome measures and covariates

The primary outcome measure was a diagnosis of mania or bipolar disorder (F30/F31) during the follow-up period. Age and gender recorded closest to the date of diagnosis of depression were included as covariates.

### Statistical analysis

The data were analysed using Stata (V.12.0).^21^ Descriptive statistics for all variables were obtained as frequencies and percentages. The association of prior antidepressant therapy and subsequent diagnosis of mania/bipolar disorder was investigated using survival analysis and multivariable Cox regression. For these analyses, the outcome variable of time to diagnosis of mania/bipolar disorder was defined as the number of years from the date of diagnosis of depression to the date of diagnosis of mania/bipolar disorder, censored at 31 March 2014. Incidence rates of mania/bipolar disorder were estimated for the overall population in the study and for subgroups defined by prior antidepressant treatment, age and gender. Unadjusted and adjusted HRs were estimated using univariate and multivariable Cox regression. The multivariable model included age, gender and prior antidepressant treatment (in the groups defined previously) in order to adjust for prior treatment with more than one antidepressant. Reference groups for age and gender in the Cox regression analysis were defined as those with the greatest prevalence for each variable.

## Results

### Incidence rate of mania/bipolar disorder

Of the 21 012 patients included in the study, 994 were diagnosed with mania or bipolar disorder during the follow-up period. The overall incidence rate of mania/bipolar disorder was 10.9 per 1000 person-years.

### Sample characteristics and association of antidepressant therapy with subsequent mania/bipolar disorder

Table 1 summarises the breakdown of demographic factors and prior antidepressant treatment within the sample and their association with subsequent mania or bipolar disorder. The majority of the sample were female and aged between 26 and 35 years, and these groups were associated with the peak incidence of mania/bipolar disorder. The most frequently prescribed antidepressants were SSRIs (35.5%), mirtazapine (9.4%), venlafaxine (5.6%) and TCAs (4.7%). All antidepressants were associated with an increased incidence of mania/bipolar disorder (unadjusted HR>1.0 for all antidepressants) with incidence rates ranging from 13.1 (TCAs) to 19.1 (trazodone) per 1000 person-years. Multivariable Cox regression analysis (adjusted for age, gender and previous antidepressant treatment) indicated a statistically significant association of prior treatment with SSRIs (HR 1.34, 95% CI 1.18 to 1.52) and venlafaxine (1.35, 1.07 to 1.70).

**Table 1:** Cox regression analysis of factors associated with mania/bipolar disorder (n=21 012)

Factor	Group	Number in sample (%)	Incidence rate of mania/bipolar disorder (per 1000 person-years)	Association with mania/bipolar disorder			
				Unadjusted		Adjusted model*	
				HR (95% CI)	p Value	HR (95% CI)	p Value
Age (years)	16–25	4586 (21.8)	10.1	0.80 (0.67 to 0.96)	0.02	0.84 (0.70 to 1.01)	0.06
	26–35	5406 (25.7)	12.3	Reference		Reference	
	36–45	5353 (25.5)	11.2	0.92 (0.78 to 1.09)	0.34	0.90 (0.76 to 1.07)	0.23
	46–55	3798 (18.1)	10.7	0.86 (0.71 to 1.04)	0.12	0.84 (0.70 to 1.02)	0.07
	56–65	1869 (8.9)	8.3	0.68 (0.52 to 0.88)	0.004	0.65 (0.50 to 0.85)	0.002
Gender	Female	12 767 (60.8)	11.1	Reference		Reference	
	Male	8245 (39.2)	10.5	0.94 (0.83 to 1.07)	0.34	0.94 (0.83 to 1.07)	0.35
Prior antidepressant treatment	MAOi	37 (0.2)	14.1	1.44 (0.46 to 4.48)	0.53	1.20 (0.38 to 3.79)	0.76
	Mirtazapine	1977 (9.4)	13.7	1.29 (1.07 to 1.57)	0.009	1.17 (0.96 to 1.43)	0.11
	SSRI	7468 (35.5)	13.2	1.38 (1.22 to 1.57)	<0.001	1.34 (1.18 to 1.52)	<0.001
	TCA	993 (4.7)	13.1	1.25 (0.96 to 1.62)	0.09	1.12 (0.86 to 2.58)	0.39
	Trazodone	160 (0.8)	19.1	1.80 (1.06 to 3.05)	0.03	1.51 (0.88 to 2.58)	0.14
	Venlafaxine	1184 (5.6)	14.9	1.46 (1.17 to 1.83)	0.001	1.35 (1.07 to 1.70)	0.01
	Duloxetine	248 (1.2)	13.8	1.27 (0.77 to 2.12)	0.35	1.10 (0.66 to 1.83)	0.73
	Other antidepressant	101 (0.5)	13.7	1.36 (0.65 to 2.86)	0.42	1.05 (0.49 to 2.25)	0.90

*Results adjusted for all the factors reported in this table.

MAOi, monoamine-oxidase inhibitor; SSRI, selective serotonin reuptake inhibitor; TCA, tricyclic antidepressant.

## Discussion

Our findings demonstrate a significant association between antidepressant therapy in patients with unipolar depression and an increased incidence of mania. This association remained significant after adjusting for age and gender.

The overall incidence of mania, independent of treatment, was 10.9 per 1000 person-years. A study by Benvenuti *et al*^22^ found an incidence rate of mania of 3.0% in patients with unipolar depression treated with SSRIs, and 0.9% in those patients treated with interpersonal psychotherapy over a 9-month follow-up period and a study in children and young adults by Martin *et al*^23^ found a rate of 5.4% over a median follow-up of 41 weeks. A recent meta-analysis estimated even greater rates of mania of 12.5% for those treated with antidepressants.^1^ These estimates from previous studies are greater than the rate found in the present study. In another retrospective study, patients with unipolar depression showed a prevalence of mania of 13.1% over a 6-year follow-up period, whereby the group which developed mania also had a higher frequency of family history of bipolar disorder than those who did not develop mania.^24^ In the present study, the HR of mania/bipolar disorder associated with antidepressant therapy ranged between 1.11 and 1.47. This compares with antidepressant-associated mania HRs of between 2.1 and 3.9 by Martin *et al*.^23^ Another study in which patients with underlying bipolar disorder were treated with antidepressant monotherapy without a mood stabiliser, the HR was found to be 2.83 vs 0.79 in patients treated with a concurrent mood stabiliser.^25^ Venlafaxine and SSRIs were consistently associated with mania/bipolar disorder in our study. These findings are in keeping with previously established associations of mania with venlafaxine,^1^ ^26–29^ as well as SSRIs.^1^ ^30–32^ It is possible that the incidence rate of mania and HR associated with antidepressant therapy in our study was lower than previous studies because the sample was drawn from patients presenting to secondary mental healthcare services. Patients presenting to mental healthcare services with unipolar depression may have already received antidepressant therapy from primary care services. Furthermore, patients may have developed symptoms of mania prior while being treated with antidepressants in primary care and would have presented to secondary care services already with an established diagnosis of bipolar disorder.

Antidepressant-induced mania has been reported more commonly in people with an established diagnosis of bipolar disorder than in people with unipolar depression.^30^ It is generally recommended that patients who have been previously diagnosed with depressive disorders while experiencing manic or hypomanic episodes on antidepressant therapy should be evaluated for bipolar disorder. The different therapeutic approach to unipolar depression versus bipolar disorder has initiated discussion on misdiagnosis in cases of patients with unipolar depression who subsequently experience episodes of hypomania or mania. Approximately half of initial episodes of bipolar disorder present initially with depression,^4^ and depressive symptoms tend to dominate the course of the illness.^5^ ^6^ In cases in which a diagnosis of bipolar disorder had been previously established, episodes of mania have been particularly associated with TCAs and venlafaxine.^3^ However, it is possible that the association of hypomania or mania with antidepressant therapy in people with a diagnosis of unipolar depression reflects an underlying bipolar depression rather than an adverse effect of antidepressants. There is ongoing debate regarding the nosological distinction between unipolar and bipolar depression^7^ ^8^ and the extent to which these two disorders can be distinguished in the absence of a prior episode of mania or hypomania.^8^

However, regardless of underlying diagnosis or aetiology, the association of antidepressant therapy with mania demonstrated in the present and previous studies highlights the importance of considering whether an individual who presents with depression could be at risk of future episodes of mania.^33^ ^34^ Apart from antidepressant therapy, other risk factors for mania or hypomania in people receiving treatment for depression include a family history of bipolar disorder, a depressive episode with psychotic symptoms, young age at onset of depression and antidepressant resistance.^35^ Although we were unable to obtain data on family history of bipolar disorder, the presence of psychotic symptoms or antidepressant resistance in our study, we did find a greater incidence of mania/bipolar disorder in patients aged between 26 and 35 years, in keeping with previous findings.^23^ Future research should not only focus on which classes of antidepressants are most associated with mania, but also on other associated factors in order to guide clinicians of the risk of mania in people with depression prior to prescribing antidepressant therapy.

There are some limitations which should be considered when interpreting the results presented in our study. Our findings are based on observational data, and so it is not possible to infer an aetiological association between antidepressant exposure and subsequent mania/bipolar disorder. The use of routinely recorded clinical data also meant we were unable to obtain data on potentially important factors such as family history of bipolar disorder, the presence of psychotic symptoms or resistance to antidepressant therapy. Our findings were based on data recorded from adults in secondary mental healthcare services. It is likely that the patients included in our study will have received a diagnosis of depression and initial treatment in primary care. It is also possible that patients who received treatment in secondary care may have been discharged back to primary care where their treatment may have been modified. Our study did not include patients who developed an episode of mania prior to receiving initial treatment in secondary mental healthcare services or prior to the age of 16 years. These patients would have been excluded from our study thereby leading to an underestimate of mania/bipolar disorder incidence. Further research is warranted to investigate clinical data recorded in patients under the age of 16 and linking data from primary care services with data from secondary care services to establish association of antidepressant therapy with mania across both clinical settings. Another limitation was the lack of available data on timing or dose of antidepressant therapy. It is possible that any association between antidepressant therapy and subsequent mania/bipolar disorder would have depended on the dose and duration of treatment, and in patients who did develop mania, how soon a particular antidepressant was given prior to the onset of symptoms of mania. Furthermore, some patients may have been switched between different antidepressants (due to lack of efficacy in treating depression) prior to onset of mania. As the antidepressant exposure in our study was determined prior to onset of mania, it is not possible to determine which antidepressants (if any) a patient was taking at the time of onset of mania.

We found an association of venlafaxine with subsequent mania/bipolar disorder. In the UK, venlafaxine is recommended as a second-line treatment for unipolar depression.^20^ It is therefore possible that this association is confounded by resistance to antidepressant therapy. Furthermore, the analysis of routine clinical records raises the possibility of confounding by indication, whereby the choice of pharmacotherapy employed by clinicians is influenced by their perception of likely beneficial or adverse effect. It is possible that other factors associated with mania could have influenced the choice of antidepressant therapy, therefore biasing our findings with respect to the observed association of antidepressant therapy with mania/bipolar disorder. This might explain why we did not elicit an association of TCAs with subsequent mania in our study despite previous studies suggesting this possibility.^1^ Another possible explanations for the lack of association of TCAs with mania in our study is their use for other clinical indications such as neuropathic pain (often at lower doses than used to treat depression) which could have reduced their association with mania.^36^

In our study, we only analysed the association of antidepressant therapy with subsequent mania or bipolar disorder. Treatment guidelines recommend that patients who do not respond to antidepressant monotherapy may benefit from augmentation with an antipsychotic or mood stabiliser.^20^ We were unable to reliably obtain data on antidepressant augmentation in our study. However, it is possible that augmentation with such agents may have affected any observed association of antidepressant therapy with the development of mania/bipolar disorder as antipsychotics and mood stabilisers have been shown to reduce the risk of developing mania^37^ and further studies are warranted to investigate the association of antidepressant augmentation on risk of mania.

Despite these limitations, we have demonstrated an association between antidepressant therapy and subsequent mania/bipolar disorder using a large data set of clinical data that is prospectively recorded and representative of everyday clinical practice in secondary mental healthcare. Our findings are therefore generalisable to people receiving standard antidepressant therapy for depression and in keeping with previous studies drawn from observational and interventional research studies. Although our findings do not demonstrate any causal link between antidepressant therapy and bipolar disorder, the association of antidepressant therapy with mania in people being treated for depression reinforces the importance of considering risk factors for mania or hypomania in people who present with an episode of depression. Our findings also highlight an ongoing need to develop better ways to predict future risk of mania in people with no prior history of bipolar disorder who present with an episode of depression.
